# Oxytocin-Gly-Lys-Arg: A Novel Cardiomyogenic Peptide

**DOI:** 10.1371/journal.pone.0013643

**Published:** 2010-10-26

**Authors:** Bogdan A. Danalache, Jolanta Gutkowska, Magdalena J. Ślusarz, Irena Berezowska, Marek Jankowski

**Affiliations:** 1 Research Centre, Centre Hospitalier de l'Université de Montréal (CRCHUM) – Hôtel-Dieu, Montreal, Quebec, Canada; 2 Department of Medicine, Université de Montréal, Montreal, Quebec, Canada; 3 Faculty of Chemistry, University of Gdańsk, Gdańsk, Poland; 4 Laboratory of Chemical Biology and Peptide Research, Clinical Research Institute of Montreal, Montreal, Quebec, Canada; University of Padova, Italy

## Abstract

**Background:**

Oxytocin (OT), synthesized in the heart, has the ability to heal injured hearts and to promote cardiomyogenesis from stem cells. Recently, we reported that the OT-GKR molecule, a processing intermediate of OT, potently increased the spontaneous formation of cardiomyocytes (CM) in embryonic stem D3 cells and augmented glucose uptake in newborn rat CM above the level stimulated by OT. In the present experiments, we investigated whether OT-GKR exists in fetal and newborn rodent hearts, interacts with the OT receptors (OTR) and primes the generation of contracting cells expressing CM markers in P19 cells, a model for the study of early heart differentiation.

**Methodology/Principal Findings:**

High performance liquid chromatography of newborn rat heart extracts indicated that OT-GKR was a dominant form of OT. Immunocytochemistry of mouse embryos (embryonic day 15) showed cardiac OT-GKR accumulation and OTR expression. Computerized molecular modeling revealed OT-GKR docking to active OTR sites and to V1a receptor of vasopressin. In embryonic P19 cells, OT-GKR induced contracting cell colonies and ventricular CM markers more potently than OT, an effect being suppressed by OT antagonists and OTR-specific small interfering (si) RNA. The V1a receptor antagonist and specific si-RNA also significantly reduced OT-GKR-stimulated P19 contracting cells. In comparison to OT, OT-GKR induced in P19 cells less α-actinin, myogenin and MyoD mRNA, skeletal muscle markers.

**Conclusions/Significance:**

These results raise the possibility that C-terminally extended OT molecules stimulate CM differentiation and contribute to heart growth during fetal life.

## Introduction

Oxytocin (OT), recognized as a female reproductive hormone, is largely produced in hypothalamic magnocellular neurons of paraventricular and supraoptic nuclei. Biochemical and recombinant DNA studies have demonstrated that it is synthesized as the non-glycosylated protein, which undergoes an initial endoproteolytic cleavage by the convertase magnolysin (EC 3.4.24.62) to OT-Gly-Lys-Arg (OT-GKR) [1], [2]. Subsequent processing produces other OT extended molecules: OT-Gly-Lys (OT-GK) and OT-Gly (OT-G) [2], often referred to as OT-X [3].

OT-G is converted by an α-amidating enzyme to C-amidated nonapeptide which is released into the circulation in this form. OT-X forms have been detected in the developing brain of animals and in fetal plasma. In rats, enzymatic OT-X conversion to OT is almost complete in adulthood, but not in fetuses, which accumulate OT-X in the brain [4], [5]. Similarly, the plasma OT-X elevation reported during early fetal development in sheep [3] is reduced in late gestation, when OT begins to predominate in the circulation.

OT's acts on only one type of OT receptor (OTR), an integral membrane protein that is a member of the rhodopsin-type (class I) G protein-coupled receptor family, which includes arginine vasopressin (AVP) receptor subtypes (V1aR, V1bR and V2). The peptide sequences of AVP and OT differ only in 2 amino acids in positions 3 and 8, which enable these hormones to interact with the respective receptors [6]. OTR and OT biosynthesis is detected in the atria and ventricles of the heart, and OT is thought to be involved in atrial natriuretic peptide (ANP) release from the cardiomyocytes (CM) of newborn rats [7], [8] and humans [9]. Indeed, OTR immunostaining of the heart is predominantly detected in CM [10]. Because radioimmunoassay (RIA) indicates OT elevation in fetal and newborn hearts at a stage of intense cardiac hyperplasia, we hypothesized a role for OT in CM differentiation [8]. Our initial experiments demonstrated that OT induces CM differentiation of the mouse embryonal carcinoma (EC) P19 cell line, a common cell model for studying early heart differentiation [11]. Several reports have confirmed OT-stimulated cardiomyogenesis in different lines of embryonic stem (ES) cells [12], [13], [14]. Some of these observations pointed to a Ca^2+^ mobilization mechanism in response to OT treatment in ES D3 cells differentiating into CM [15]. We established that the OTR-nitric oxide-cGMP pathway is essential for the OT-elicited differentiation of P19 stem cells into CM in association with elevation of transcription factors GATA-4 and myocyte-specific enhancer factor 2c (Mef2c) [12]. More recently, we obtained evidence that OT-GKR possesses biological activity. Since treatment with OT-GKR stimulated glucose uptake in rat CM [16] and enhanced spontaneous cardiomyogenesis of ES D3 cells more potently than OT nonapeptide [15].

In the present study, we reasoned that if OT-GKR plays a role in cardiomyogenesis we should detect the molecule in fetal and newborn animal hearts. To investigate whether the biological actions of OT-GKR are mediated by the OTR, the interactions of these molecules were analyzed by computer-generated 3D models. Because EC P19 cells do not spontaneously differentiate into CM after their aggregation in the presence of fetal calf serum [11], as has been observed for ES D3 cells [15], therefore we used these cells to analyze whether OT-GKR treatment generate the contracting colonies expressing CM markers. The specificity of this reaction was examined by inhibition by OT antagonists (OTA) and OTR suppression by specific small interfering RNA (siRNA) and similar condition was investigated for V1aR.

## Results

### OT-GKR is the dominant OT form in developing hearts

Synthetic OT-X molecules and OT were used to identify elution profiles from the HPLC column. Specific wavelength intervals at 215 and 280 nm were calibrated by spectrophotometry. Subsequently, HPLC-specific retention factor for OT standards was k' = 0.67 for OT, k' = 1.34 for OT-GKR, k' = 1.67 for OT-GK, and k' = 2.01 for OT-G. Similar retention factors of these molecules have been disclosed in heart extracts from newborn rats. Analysis of eluates, released from the column, revealed a small peak at k' = 0.67, corresponding to OT, and a large second peak at k' = 1.34, calibrated as a retention factor of OT-GKR. A minor peak was observed at k' = 1.67, the point of OT-GK elution, and a minimal peak, if any, was detected at the point of elution of OT-G. The presence of OT-GKR in specific HPLC fractions was confirmed by specific RIA with antibodies specific for OT and antibodies detecting OT-X forms.

### Immunocytochemistry reveals OT-GKR in developing hearts

**Figure 1:**
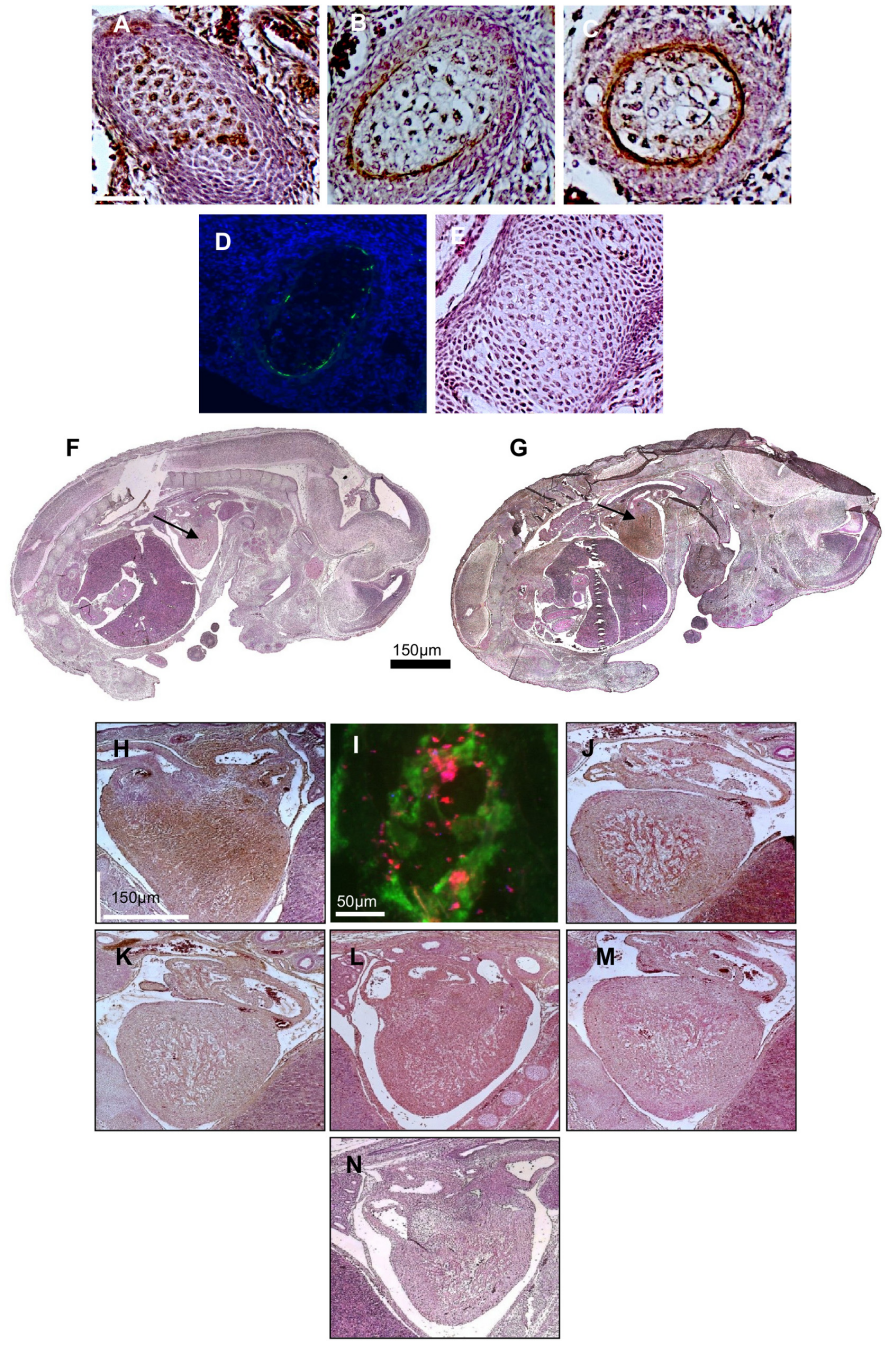
Detection of the OT system in the E15 mouse embryo by immunocytochemistry. Somite staining with OT-GKR antibody (**A**) and selective antibodies for OT (**B**) and OTR (**C**). TUNEL reaction displaying apoptosis (**D**)**.** Control staining of somites with OT-GKR-specific antibody reabsorbed with OT-GKR (**E**) Control staining of whole embryo with OT-GKR-specific antibody reabsorbed with OT-GKR (**F**). Immunodetection of OT-GKR in the fetal mouse heart at day E15 (arrow) (**G**). Polyclonal rabbit antibody specifically recognizing OT-GKR peptide was applied to detect OT-GKR. Staining was revealed by the biotin-streptavidin method. Higher magnification of OT-GKR staining in the fetal heart (**H**). Immunofluorescence of OT-GKR (Texas Red) in cryostat sections section of cardiac tissue (stained green by troponin Alexa Fluor 488 antibody) by (**I**)**.** Immunodetection in the heart of OTR (**J**)**,** OT (**K**), AVP (**L**), and V1R (**M**)**.** Control staining with OT-GKR-specific antibody reabsorbed with OT-GKR (**N**).

Immunocytochemistry of whole mouse embryos (embryonic day 15) demonstrated the entire OT system in somites, mesoderm masses distributed along neural tubes and developing into the dermis, skeletal muscle and vertebrae. As shown in Figure 1A, significant OT-GKR expression was found inside, whereas OT nonapeptide (Fig. 1B) and OTR (Fig. 1C) were viewed on the periphery of somites showing apoptosis (Fig. 1D). Control staining with OT-GKR-specific antibody, pre-absorbed with OT-GKR, was negative in somites (Fig. 1E) as well as in whole mouse sections (Fig. 1F). In sections stained with OT-GKR antibody, intense brown staining was observed in fetal hearts (Fig. 1G and 1H). Double staining by immunofluorescence indicated OT-GKR deposits in cells stained with the CM marker troponin C (Fig. 1I). Fetal heart sections were also stained with anti-OTR antibody (Fig. 1J) and with anti-OT antibody the staining was barely visible (Fig. 1K). Immunostaining with anti-vasopressin (Fig. 1L) was negative and some staining was found with anti-V1aR antibody (Fig. 1M). No staining was seen in the corresponding negative control (Fig. 1N).

### Docking analysis shows OT-GKR interaction with OTR

**Figure 2:**
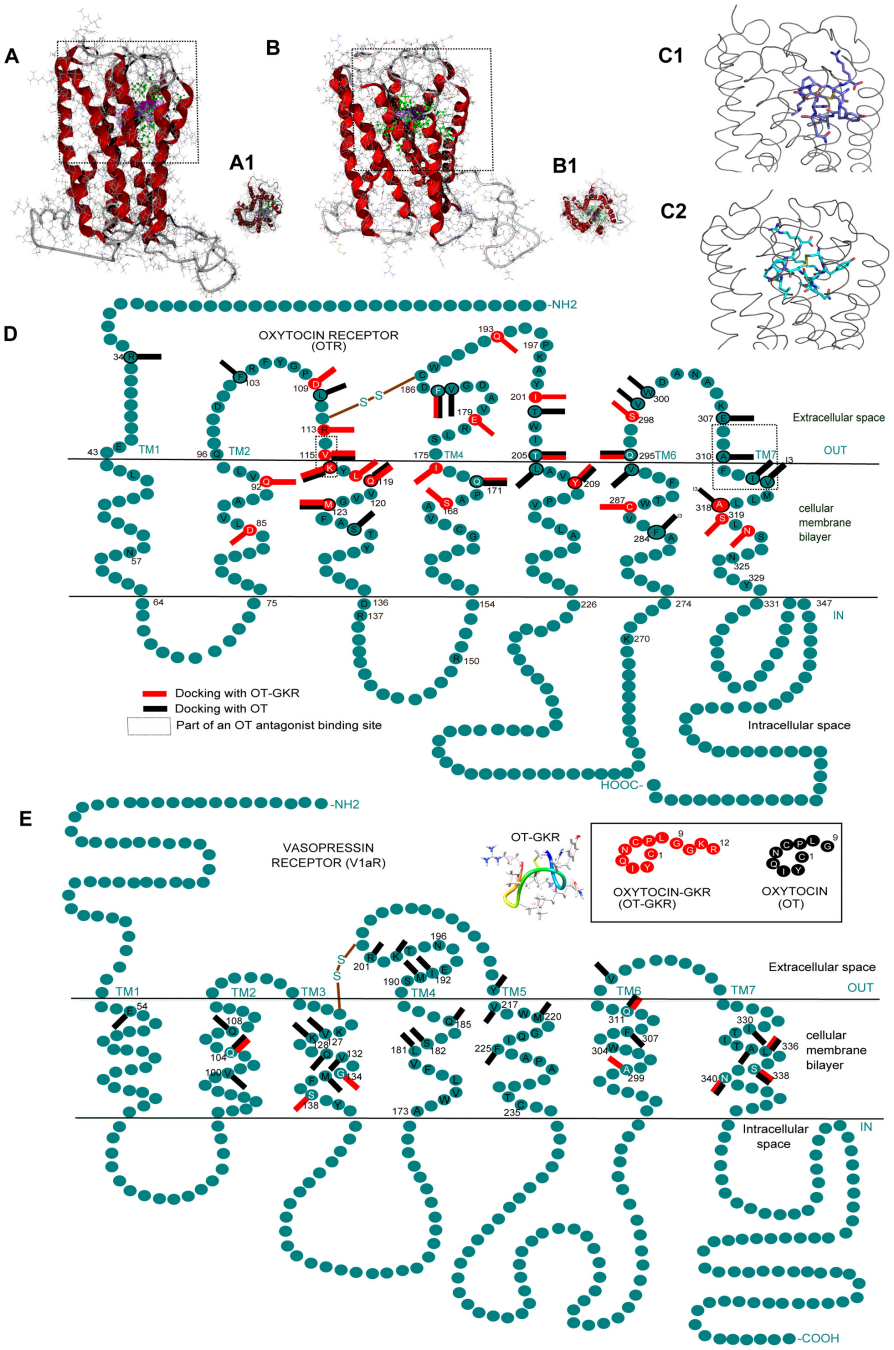
Molecular docking of 3-D models of activated human OTR and V1aR with OT/OT-GKR peptides obtained by the MolDock Optimizer algorithm from Molegro Virtual Docker. (**A**) The front upright view position (side view) of the OTR-OT- GKR complex structure. (**A1**) The section (rectangle) shown in panel A1 from the top a intracellular view (i.e. rotation by 90° out of plane) of the marked section in A, demonstrate OT-GKR (green) in active conformation inside the OT binding site (the transmembrane helices in red and the cavity in violet). (**B**) side view of the V1aR-OT- GKR complex, (**B1**) V1aR top view. (**C1**) detail of docking view of OTR-OT-GKR complex, and (**C2**) detail of V1aR-OT-GKR complex. **D** displays the schematic model of human OTRs with marked amino acid residues that are putatively involved in ligand-binding. The amino acid residues in black circles have been proposed as OT docking sites, and the red bars represent docking sites of OT-GKR. (**E**) Schematic model of human vasopressin V1aR binding with OT-GKR and OT. Amino acid residues are identified by a 1-letter code in Table 1.

Because of significant molecular differences between OT-GKR and OT, the question has been raised whether OT-GKR interacts with OTR binding sites. For this reason, we performed computational docking analysis of OT molecules in OTR binding sites. Figure 2 illustrates the docking of 3-D human OTR with OT-GKR and OT-modeled molecules in front upright view (Fig. 2A) and a view from the extracellular side (Fig. 2B). Three conformations for both OT-GKR and OT were analyzed. Six related receptor-Gα-segment-OT-GKR complexes were obtained. Possible hydrophobic and electrostatic interaction points in dynamic complexes of these molecules were indicated by estimated binding affinity energies of −6.6±0.4 kJ/mol for OT-GKR and −11.8±0.6 kJ/mol for OT. Using distance criteria, the program identified receptor amino acid residues interacting with ligands. The essential hydrogen bond and strong electrostatic interactions between both OT molecules and the receptors were characterized by visual inspection. The results are reported in Figure 2D and Table 1. Several amino acid residues have been proposed to interact with OT and OT-GKR in the OTR model where the red bars represent docking with OT-GKR, and the black bars indicate docking with OT molecule (Fig. 2D). Docking to OTR in positions V115, K116, Q119, M123, Q171, F185, T205, Y209 and Q295 was noted for both OT and OT-GKR models (Table 1). These docking positions constituted 42% of all observed docking sites of OT-GKR. Among OT-GKR interactions, the special notice should be given to binding of the arginine-12 (R12), (Fig. 2 D) to OTR. We also analyzed the docking of OT-GKR and OT molecules in the 3-D human V1aR model molecules were similar binding affinity energies of -7,38±0.3 kJ/mol for OT-GKR and −11,11±0.6 kJ/mol for OT. Figure 2E and Table 1 show OT-GKR and OT docking at respective binding sites. Both molecules were docked in positions of Q104, K128, Q311, L335, S338 and N340. The OT-GKR was bound exclusively in positions of G134, S138 and A299.

**Table 1:** List of the OTR, V1aR residues, involved in the List of the OTR interactions with OT-GKR, and OT; * - AVP agonist binding site; ** - OTA antagonist binding site.

OTR			V1aR		
	OT	OT-GKR	OT	OT-GKR	
**TM2**	-	D85	V100	-	
	-	Q92	Q104	Q104*	**TM2**
	V115**	V115	Q108	-	
	K116**	K116	V127	-	
	Q119	Q119	K128	K128	
**TM3**	-	L118	Q131	-	
	M123	M123	V132	-	**TM3**
	-	F124	-	G134	
	S126	-	M135	-	
	-	S168	-	-	
**TM4**	Q171	Q171	-	S138	
	-	I174	L181	-	
	-	E179	S182	-	**TM4**
	V184	-	Q185	-	
	F185	F185	Y216	-	
	-	Q193	V217	-	**TM5**
	-	I201	M220	-	
	T202	-	F225	-	
	T205	T205	-	A299	
	L206	-	F307	-	**TM6**
**TM5**	Y209	Y209	Q311	Q311*	
	Q295	Q295	I330	-	
	V294	-	A334	-	
**TM6**	-	C287	L335	L335	**TM7**
	F284	-	S338	S338	
	A310**	-	N340	N340	
	I313**	-			
	V314**	-			
**TM7**	A318	-			
	-	S319			
	-	N321			

### OT-GKR stimulates contracting cell colonies in EC P19 cells

**Figure 3:**
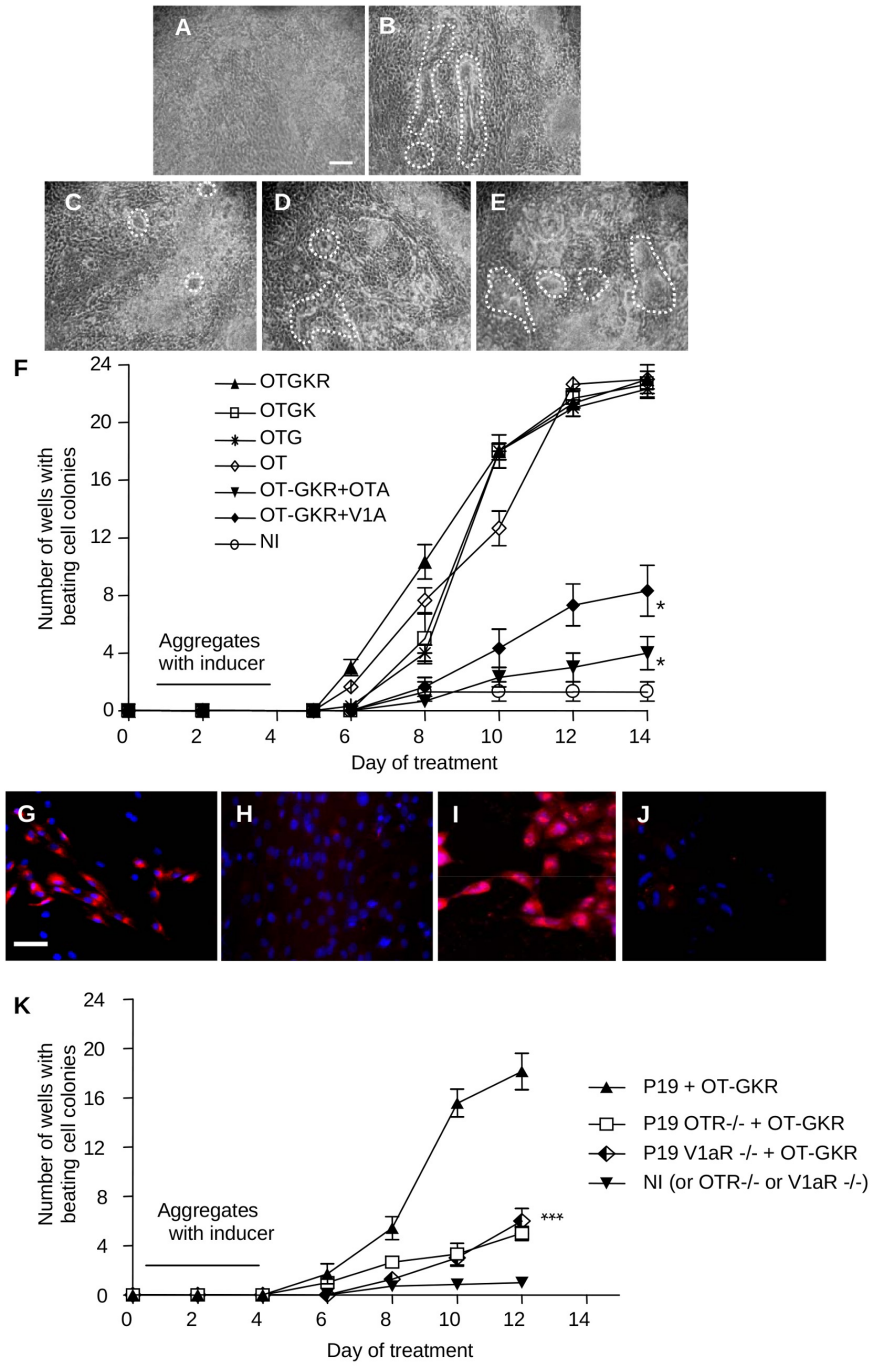
Generation of beating cell colonies of EC P19 cells after induction with different OT forms. Micrographs (100× magnification) show cultures at day 10 with dotted lines encircling beating colonies and induced by: (**A**) Non-inducer, (**B**) OT, (**C**) OT-G, (**D**) OT-GK, (**E**) OT-GKR. (**F**) Time course of appearance of beating cell colonies. At day 5, cultures were transferred into a 24-well tissue culture dish and scored every 2 days for the presence of beating cell colonies. The generation of beating cell colonies was induced by treatment of EBs (from day 1 to 4) with OT, OT-G, OT-GK or OT-GKR in the presence of antagonists for OTR and V1aR (see Materials and Methods). The results are expressed as means ± SEM of 3 independent experiments. All treatments produced significantly different numbers of beating cell colonies than non-induced controls (NI). **G** demonstrates OTR immunodetection in cells at day 2 in control EC P19 cultures and (**H**) in cells subjected to OTR^−/−^ siRNA treatment**,** (**I**) detection of V1aR in control**,** and (**J**) in these cells subjected to V1aR^−/−^ siRNA treatment. (**K**) OTR and V1aR inhibition by siRNA in EC P19 cells decreases their ability to generate beating cell colonies after induction with OT-GKR (10^−6^ M). *p<0.05, NI – not induced control.

EC P19 cell cardiogenic differentiation is not spontaneous or very rare. With the hanging drop method in the presence or absence of inducers, EC P19 embryoid bodies (EBs) were analyzed for their beating activity from day 2 until 14 after plating. In cell cultures not exposed to inducers (NI) no beating cell colonies were found (Fig. 3A), although they sometimes displayed rare beating foci. We chose micromolar concentrations to compare the action of OT and OTX forms. The 10^−6^ M concentration was found to be the most efficient in inducing cardiomyogenesis. As with OT treatment, extended OT forms induced the appearance of numerous colonies, with cell-specific organization, in linear, parallel arrays or round clusters displaying synchronized contractions (Fig. 3B-E). Videoanalysis revealed that OT and OT-X were equally efficient in producing beating cell colonies by day 14 (Fig. 3F). However, observations on day 8 disclosed a significantly larger number of contracting cell colonies in samples induced by OT-GKR (9±0.6) compared to cells induced by OT (5±0.4), OT-G (4±0.6) or OT-GK (7±0.6), p<0.05.

To ascertain OTR involvement in OT-GKR-mediated cardiomyogenesis, OT-GKR-treated EBs were additionally exposed to 10^−6^ M OTA (Fig. 3F). Indeed, OTA effectively inhibited the number of OT-GKR-stimulated contracting colonies (2±03 foci in 24 wells at day 8, and 4±0.6 at day 12) *vs.* OT-GKR (23±0.2/24 at day 12, p<0.05). Application of V1R antagonist also diminished the number of beating cells induced by OT-GKR (to 2±03 foci in 24 wells at day 8 and 8±0.6 at day 12, p<0.05), indicating that both OTR and V1aR contribute to the generation of contracting cell colonies.

To verify receptor function in the cardiomyogenesis of EC P19 cells, we used specific siRNA for OTR and V1aR silencing. As illustrated in Figure 3G, OTR staining with OTR antibody disclosed OTR expression in most EC P19 cells. Similar staining for V1aR was presented in the Fig. 3I. This was significantly reduced in cells subjected for the both siRNA treatments (OTR - Fig. 3H, respectively V1aR - Fig. 3J). Consequently, in siRNA-treated cells at day 12, OT-GKR treatment resulted in the production of very small clusters of weakly beating cells in only 4±0.2 of 24 wells whereas 23±0.2 control wells displayed beating cell colonies (Fig. 3I), p<0.05. Application of siV1aR also diminished the number of beating cells induced by OT-GKR (to 2±0.2 foci in 24 wells at day 8, 6±1.0 at day 12, p<0.05) indicating that both OTR and V1aR contribute to generation of contracting cell colonies and CM differentiation.

**Figure 4:**
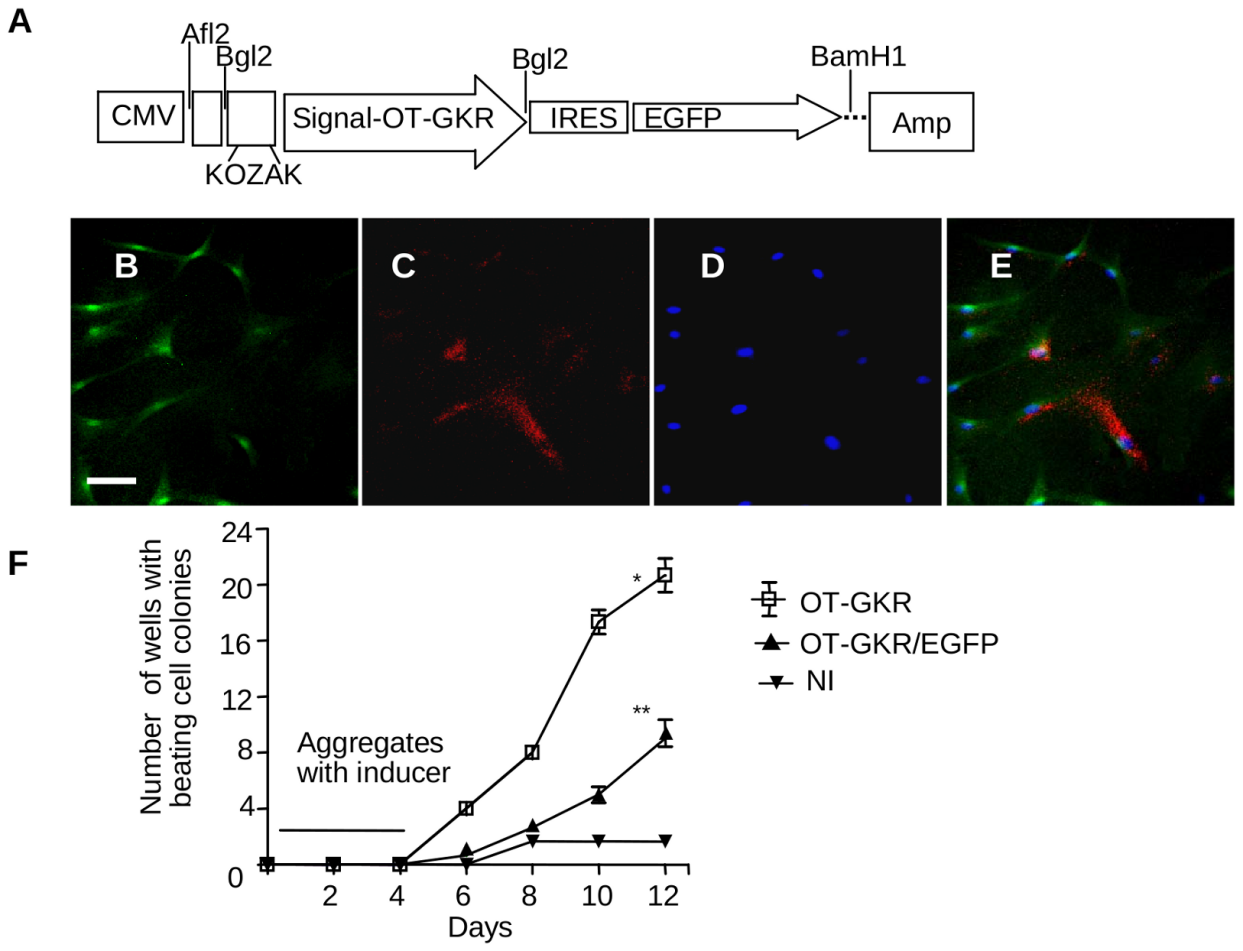
Schematic structure of the OT-GKR-IRES-EGFP DNA construct. Abbreviations: CMV, cytomegalovirus; EGFP, enhanced green fluorescent protein; OT, oxytocin. (**A**) pcDNA3.1/Amp-OT-GKR-IRES-EGFP transfection to EC P19 cells stimulates the expression of green fluorescence (**B**) and produces OT-GKR protein marked with VA-18 antibody in red (Texas Red) **(C)**. Blue DAPI staining of cell nuclei (**D**) and merged photo (**E**). Time course of appearance of beating cell colonies in non-induced EC P19 cells transfected with OT-GKR-IRES-EGFP and in normal EC P19 cells stimulated with OT-GKR (10^−6^ M), and non-induced controls (NI) (**F**).

Because OT-GKR accumulation in fetal hearts was seen inside cells expressing the CM marker, we investigated whether the endogenous production of OT-GKR in EC P19 cells initiates cardiomyogenesis. For this purpose, the cardiogenic potency of OT-GKR was studied in EC P19 cells stably expressing the pcDNA3.1/Amp-OT-GKR-IRES/EGFP (green fluorescence) construct (Fig. 4A). As shown in Figure 4B-E, OT-GKR protein was disclosed by immunofluorescence in approximately 30% of transfected cells. Although at day 12, the beating cell colonies induced by endogenous OT-GKR were fewer (10/24±0.4) than those receiving OT-GKR from the medium (21/24±0.6, p<0.05), the OT-GKR-transfected cells displayed large clusters of beating activity (Fig. 4F).

**Figure 5:**
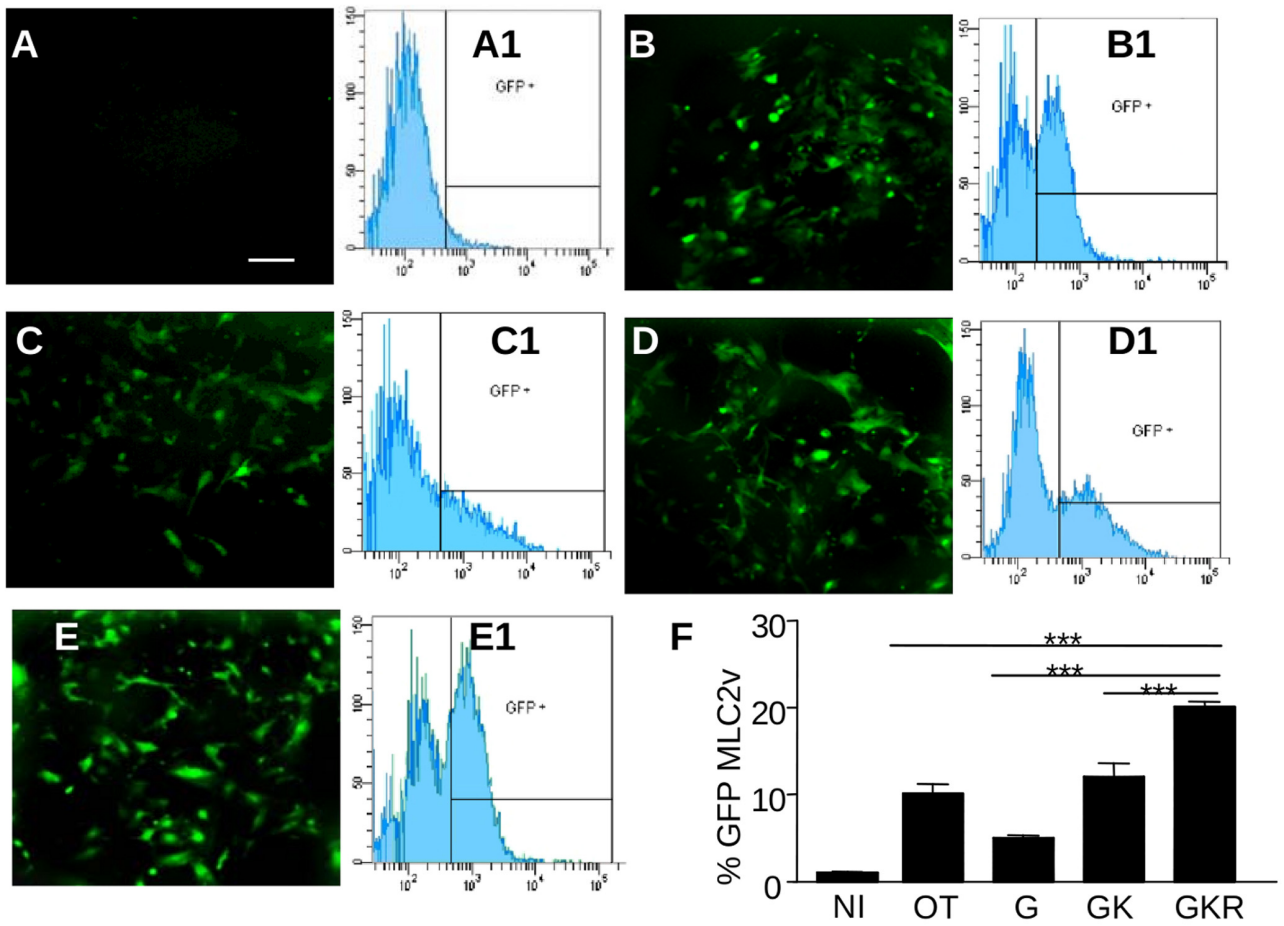
In response to treatment with OT or OT-X forms, GFP-P19Cl6 cells express green fluorescence driven by the transcriptional promoter of myosin light chain-2 ventricular (MLC-2v). Fluoromicrograph (100× magnification) of day 8 cell cultures in non-induced conditions (**A**) and upon induction with OT (**B**), OT-G (**C**), OT-GK (**D**) or OT-GKR (**E**). Representative flow cytometry charts of fluorescence emitted by GFP-P19Cl6 cells at day 6 in non-induced conditions (**A1**) and in cultures induced by OT (**B1**), OT-G (**C1**), OT-GK (**D1**), and OT-GKR (**E1**). Bars present quantitative analysis of fluorescence emitted by differently-stimulated GFP-P19Cl6 cells at day 6 in 3 independent experiments (**F**).

For the quantitative assessment of cardiomyogenic differentiation, the EGFP expression pattern in P19Cl6-GFP cells was analyzed by fluorescence microscopy and flow cytometry. Figure 5 characterizes the cells derived from EBs at day 8, representing the early cardiomyoblast stage. Brilliant GFP fluorescence indicated the expression of the specific ventricular marker MLC-2v. At day 8, GFP-positive areas were absent in NI cells (Fig. 5A), whereas cells induced by OT (Fig. 5B) and OT-X (Figs. 5C-E) exhibited identical, intense GFP fluorescence. However, flow cytometry analysis (illustrated in Figs. 5A1-E1) demonstrated a significantly larger number of GFP-positive cells upon OT-GKR stimulation in comparison to stimulation with OT, OT-G or OT-GK (Fig. 5F, p<0.05).

### OT-GKR induces differentiation markers in EC P19 cells

**Figure 6:**
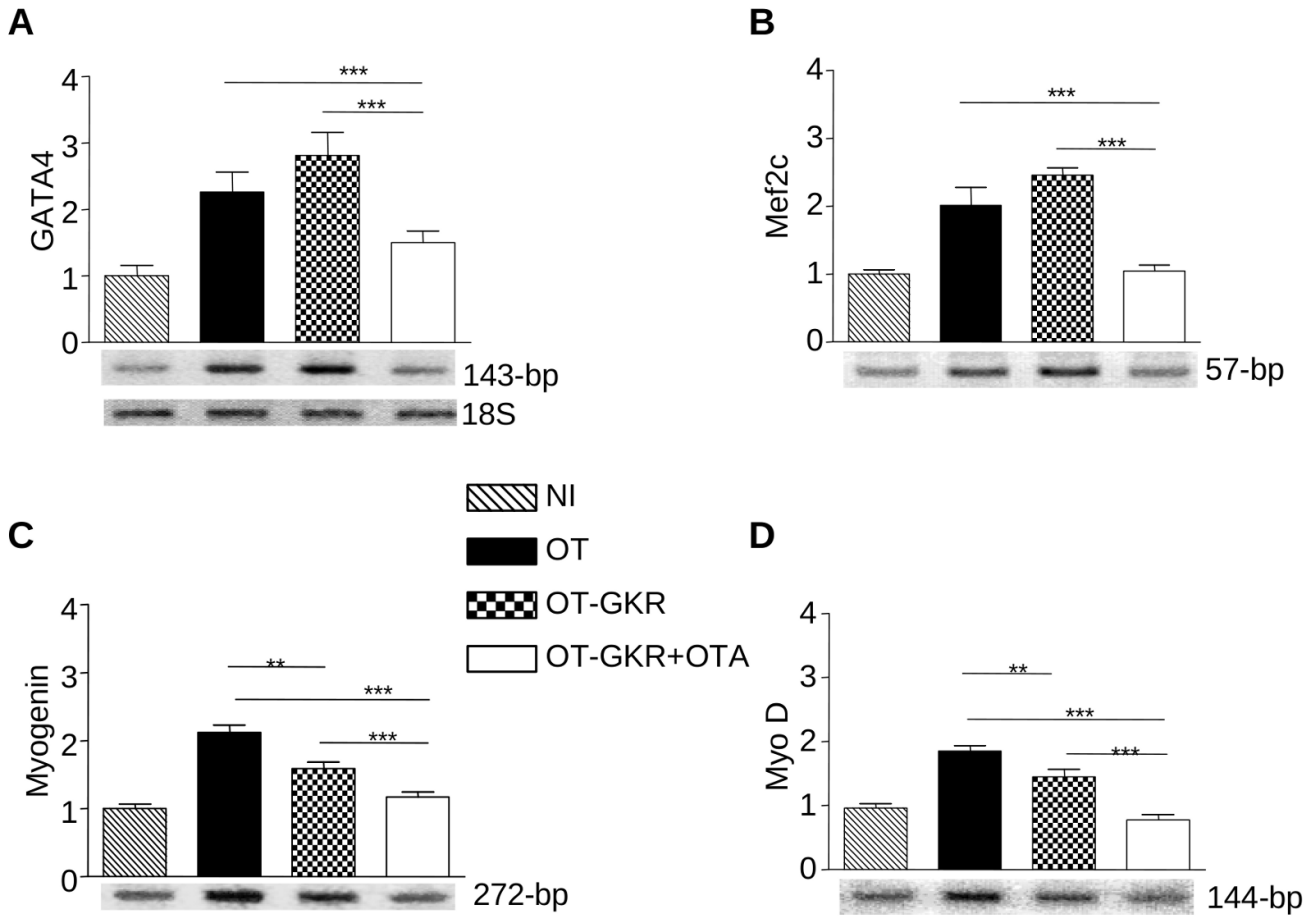
RT-PCR analysis of GATA-4 (A), Mef2c (B), myogenin (C) and MyoD (D) transcripts in EC P19 cells induced by OT or OT-GKR in the presence or absence of OT antagonist (OTA). NI indicates non-induced controls.

Cardiac and skeletal markers were altered during OT-mediated cell differentiation. Figure 6 illustrates the expression of genes at the 6^th^ day of EC P19 cell differentiation, when the first beating cell colonies were detected. Exposure of EC P19 cells to OT and OT-GKR increased GATA-4 mRNA (Fig. 6A), the transcription factor involved in cardiac development, and Mef2c mRNA (Fig. 6B), the gene involved in cardiac morphogenesis and myogenesis. As shown in Figures 6C and 6D, treatment also induced the mRNA genes involved in skeletal muscle morphogenesis, myogenin mRNA (Fig. 6C) and MyoD mRNA (Fig. 6D). Interestingly, the expression of these markers was higher in OT-induced cells than in cells stimulated by OT-GKR. Induction of all tested mRNA by OT-GKR was reduced in the presence of OTA to the level seen in NI controls (Fig. 6A-D).

**Figure 7:**
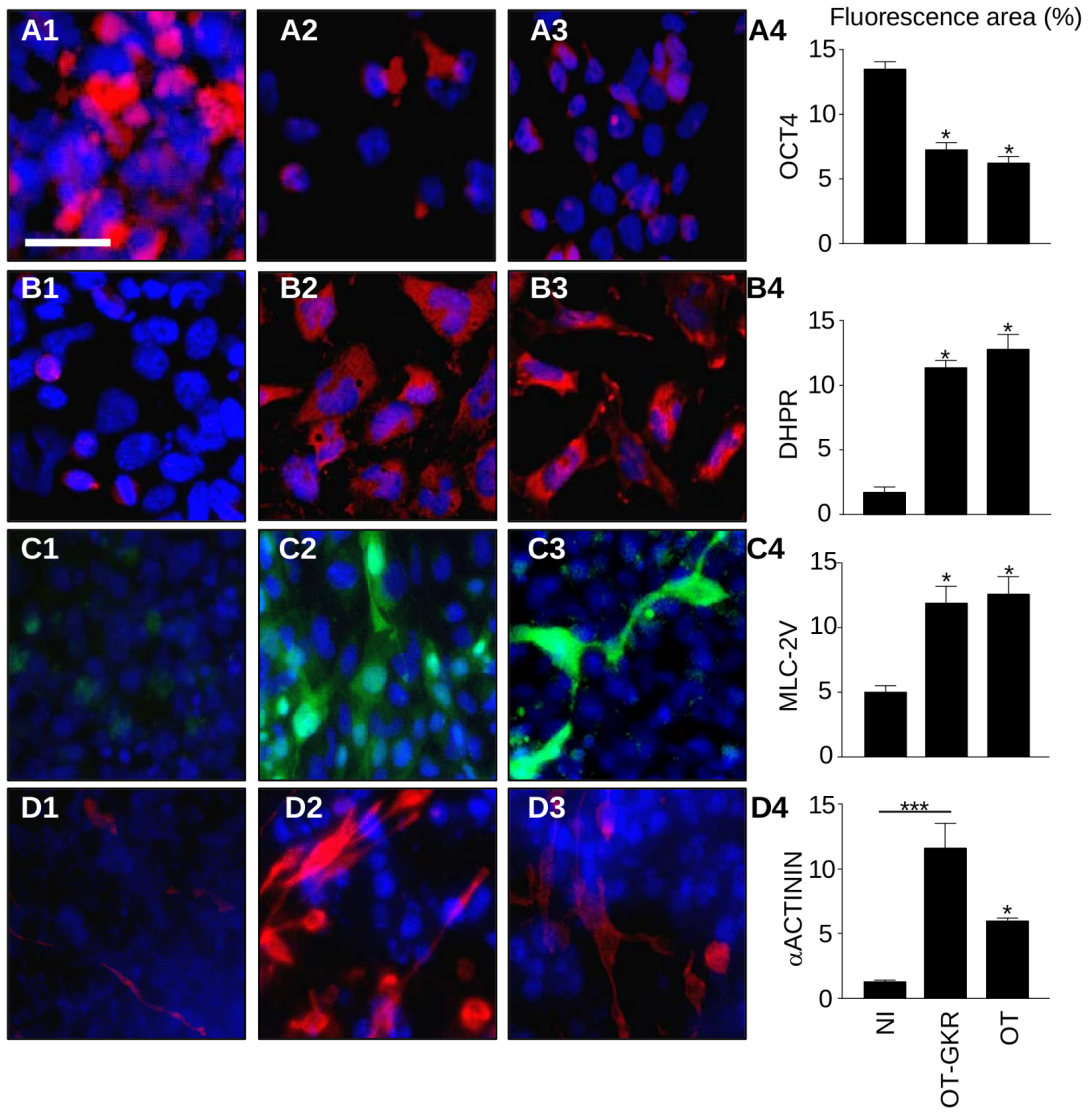
Immunofluorescence of cell differentiation markers in EC P19 cells of non-induced controls (A1-D1) and in cells stimulated to differentiate with OT (A2-D2) and OT-GKR (A3-D3). (**A4**) Staining of OCT-4, a marker of the undifferentiated state. (**B4**) Expression of the cardiac muscle marker dihydropyridine receptor-α1 (DHPRα1). (**C4**) Ventricular myosin light chain-2 ventricular (MLC-2v) protein. (**D4**) Immunocytochemistry shows staining of α-actinin protein in cardiac and skeletal muscles. **A4-D4** represent quantitative analysis of corresponding markers expressed as fluorescent areas. Fluoromicrographs of cells on differentiation day 6 presented at ×100 magnification.

The differentiation process in cells induced by OT and OT-GKR was indicated by loss of OCT-4 immunostaining, a marker of the undifferentiated state (Figs. 7A-A4). Cells treated with OT (Fig. 7B2) or OT-GKR (Fig. 7B3) displayed DHPRα markers of the advanced contractile apparatus and cardiac transcription factor MLC-2v, marker of the ventricular phenotype (Figs. 7C2 and 7C3). Sarcomeric α-actinin, the marker of both skeletal and cardiac muscles, was produced in larger quantities by cells treated with OT (Fig. 7D2) than those stimulated with OT-GKR (Fig. 7D3).

## Discussion

This study reports original observations that: (i) Fetal mouse and newborn rat hearts produce the OT-GKR molecule in cells expressing the CM marker troponin C; (ii) Experiments on EC P19 cells, the model of early heart differentiation, demonstrate the induction of beating cell colonies and CM markers in cell aggregates treated with OT-GKR protein or in cells transfected with the DNA construct carrying OT-GKR sequences; (iii) Computer modeling indicates OT-GKR interaction with the model of OTR *via* several amino acid residues, overlapping in 25% of OT binding sites; (iv) The inhibition of OT-GKR cardiomyogenesis in EC P19 cells by OTA and OTR-specific siRNA further confirms the role of OTR; (v) The interaction of OT-GKR with the V1aR model and the partial inhibition of OT-GKR-mediated stimulation of beating cell colonies in EC P19 cells by AVP antagonist and V1aR-specific si-RNA suggest that the OT-GKR can bind to and activate V1aR as well as OTR.

Our previous data indicated that extended OT forms could be produced in the developing heart since OT synthesis was seen in CM cultures from newborn rats [8] and in EC P19 cells [12]. In the present study, HPLC analysis of newborn rat hearts and immunocytochemistry of whole mouse embryos revealed that OT-GKR is abundant in developing rodent hearts. The selectivity of OT-GKR antibodies and the lack of their reaction with OT nonapeptide have already been demonstrated by RIA cross-reactivity analysis [15]. Moreover, confocal microscopy in D3 stem cells producing OT-GKR from transfected cDNA constructs further indicated positive reactions with anti-OT-GKR but negative reactions with anti-OT antibodies [15]. On the other hand, recent data suggest the positive effects of OT on osteoblast development, together with bone formation defects of OT and OTR deficiency [17]. In the present study, localization of the OT system in somites, embryonic vertebral precursors, is consistent with findings that OT is involved in osteogenesis and is important in skeleton mineralization [17], [18]. This result provides further evidence that identification of the OT system in fetal hearts is specific and not due to methodological aberrations.

We have already reported that OT-GKR increases cytosolic Ca^2+^ in ES D3 cells [15]. Among receptors of neurophyseal hormones, this effect is attributed to OTR and V1aR, whereas V2R is coupled with adenylate cyclase and the second messenger cAMP [6]. Moreover, all of these receptors have the ability to bind AVP and/or OT with varying affinities. Thus, both ligands are capable of initiating signaling cascades mediated by either receptor [19]. In fact, our studies indicate involvement of NO synthases in the CM differentiation mediated by OT [12] as well as AVP [20]. This suggests physiological relevance of both OT and AVP systems and its versatility in the cardiomyogenesis. Early studies had demonstrated that eNOS favors the maturation and cardiomyogenesis of murine embryoid bodies in vitro, because chronic NOS inhibition in these embryonic cells with the guanylyl cyclase inhibitor, ^1^H-[1], [2], [4]oxadiazolo-[4,3-a]-quinoxalin-1-one (ODQ), resulted in differentiation arrest; the event reversed upon incubation with the NO donor [21]. Likewise, NO donors and human iNOS-gene adenoviral transfection in mouse embryonic stem cells cultured in embryoid bodies facilitated their differentiation into beating cardiomyocytes [22]. OT signaling targeting eNOS is generally activated through a PLC/calcium/calmodulin pathway [23], but eNOS activation may also occur via the phosphatidylinositol-3-kinase (PI-3-K)/AKT pathway [24] as has been found in endothelial cells [25] and in CM [16], [26]. This raises the possibility of NO being a critical OT signaling molecule for both the preservation of cardiac cells and the differentiation of cardiac stem cells reserve. In P19 cells expressing GFP under a cardiac-specific promoter to monitor their CM differentiation, NO stimulated guanylate cyclase to produce cGMP, an activator of cGMP-dependent protein kinase G [12]. Recent study indicate that NO pathways that promote CM differentiation include repression of self-renewal genes, such as NANOG, and increase in differentiation genes such as GATA4 [27]. During proliferation of HUVEC, calcium mobilization in response to OT treatment seems to be instrumental in the activation of the NO pathway, as shown by the dramatic reduction of OT-induced NO release when calcium is chelated [28]. We demonstrated that in CM, chelation of intracellular calcium by BAPTA, and inhibition of calmodulin kinase II dramatically reduced the OT-mediated glucose uptake [16]. Ca^2+^ mobilization study in D3 cells showed functional OT-GKR and OT activity in embryonic stem cells [15]. The observed sustained effect on Ca^2+^ may be due to the nature of both peptides. In support of this notion, prolonged and long-lasting Fura-2 mobilization of Ca^2+^ was demonstrated in cardiac cells in response to AVP [29], [30]. Further data suggest that an activation of OTR provide a local Ca^2+^ signal that induces eNOS activation [15] and possibly natriuretic peptides [11]. By comparison, the guanylyl cyclase receptors of natriuretic peptides generate cGMP that can also contribute to CM differentiation from embryonic stem cells [31]. The increase in the mRNA expression of the cardiac-specific transcription factor Nkx2.5 and cardiac markers MLC2 and MHC followed treatment of embryonic stem cells with NO donors and cGMP activators [32]. In our previous studies in P19 cells, the GATA4 expression was only moderately reduced when l-NAME was administered together with OT, but the transcription factors MEF2c and Nkx2.5 were extensively downregulated. This lack of balance of transcription factors can severely impair the cardiomyogenic program, which requires physical interaction and synergistic modulation of target gene expressions [33], [34]. The differences in the expression of myogenic regulatory factors, MyoD and myogenin, as well as GATA4 and MEF-2, in response of P19 cells to OT and OT-GKR stimulation, can influence the developmental decisions of stem cells differentiating into the skeletal or cardiac muscle lineage. Indeed, the difference α-actin expression in OT and OT-GKR-differentiated P19 might be directly related to the level of myogenic regulatory factors promoting skeletal muscle lineage [35].

We performed computerized docking analysis to assess the relationship between OT-GKR, OTR and V1aR. Interactions between OTR residues and 4 OT-GKR amino acids Ile-3, Leu-8, Tyr-2 and Arg-12 were observed in OTR-OT-GKR complexes. The recognition of Ile-3 by OTR seems to be specific for OT molecules, because this amino acid residue is absent in the cyclic part of the AVP molecule. Replacement of Ile 3 by other amino acid residues causes a significant decrease of affinity for OTR [36]. Usually, the OT-binding site is formed by transmembrane helices 3-7 and extracellular loops 2 and 3 of the receptor [37]. Based on studies on other members of the OT-VP receptor family, specifically the V1aR, it is hypothesized that that the cyclic part of OT is lodged in the upper third of the receptor binding pocket and interacts with transmembrane domains 3, 4, and 6, whereas the linear C- termini part of the OT molecule remains closer to the surface and interacts with transmembrane domains 2 and 3, as well as with the connecting first extracellular loop [6]. This hypothesis is supported by various findings using site-directed mutagenesis techniques, as well as by domain swapping experiments between the OTR and the V2R [6], [19]. In our study, molecular docking of OT and OT-GKR showed that while both peptides are able to interact with OTR with significant binding energies, the binding pocket for OT-GKR might be slightly different from the binding pocket for OT. The results demonstrate that OT-GKR can interact with several sites in transmembrane domains 3, 4, 5 and 7 of V1aR. OT binding sites disclosed in the V1aR model are in total agreement with those reported by Ślusarz et al. [38]. These results provide guidelines for experimental site-directed mutagenesis and if confirmed, they may be helpful in designing new selective OT analogs with agonistic properties for OTR and V1aR.

Some data suggest that OT-GKR binding to V1aR is functional. V1aR is present in ES D3 cells in the very early stages of cardiac development and is then strongly down-regulated [20]. The inhibition of P19 cells differentiation involving OTA revealed that it did not reduce the number of OT-GKR-stimulated beating cell colonies to control levels. This indicates the presence of other signaling pathways in response to OT-GKR. The high sequence identity between OT and AVP receptors [39], suggest that AVP receptors may be involved, at least in part, in OT-GKR-mediated pathways. We observed that both V1aR antagonist as well as V1aR silencing partially blocked the cardiomyogenic effects of OT-GKR in EC P19 cells. The results are consistent with the observation that OTA completely blocked OT-mediated stimulation of glucose uptake in rat neonatal CM, whereas the glucose uptake induced by OT-GKR was only partially blocked [16].

Extended C-terminal OT peptides stimulate EC P19 cell differentiation into beating cell colonies expressing CM markers. OT-GKR displays the highest cardiomyogenic action among OT molecules. Both OT and OT-GKR treatment of EC P19 cells and their derived clone, EC P19 Clone 6, expressing a GFP reporter under the transcriptional control of the MLC-2v promoter, produced similar morphological changes and induced GFP fluorescence. For the moment, this action relates to differentiation to the ventricular CM phenotype since the GFP-P19Cl6 model of CM differentiation is controlled by the ventricle-associated MCL-2v promoter [40]. Nevertheless, the potential of OT and OT-GKR to promote the ventricular phenotype is of direct interest in the development of cell therapies for the heart. Already known is a positive effect of OT/OTR in inflammation [41], [42], [43]. Furthermore, recently, OT treatment had a beneficial effect in healing myocardial infarction [26], [43], [44]. The clinical application of OT-GKR in this pathology could be safe because of the specific interaction with OTR and V1aR, as described in the present study. The weaker effects of OT-X than OT on uterine contractions have already been reported [45], and OT replacement by OT-GKR in the therapy of cardiac pathologies could reduce vasoconstriction attributed to V1aR activation by OT [46].

A specific question is whether the cardiomyogenic action of OT-X is the result of cleavage to the site of OT peptide by proteolytic activity potentially present in EC P19 cultures. In a study by Altstein et al. [4], [5], however, rat brain and plasma pro-AVP cleavage efficiency in adults and fetuses was high (99 and 95% cleavage, respectively), resulting in the formation of fully processed amidated AVP forms, with no detectable, partially-processed peptides. Pro-oxytocin (pro-OT) processing in adults was very similar (over 99% cleavage), eliciting the formation of fully-processed, amidated OT. However, pro-OT processing efficiency in the fetus was very low and incomplete, culminating in 40% unprocessed precursor and the accumulation of C-terminally extended OT-X. In the same line of reasoning, it is possible that OT-GKR exhibits different efficiencies compared to OT with respect to other OT functions in the heart, such as stimulation of ANP release [47]. If this is the case, we can speculate that the relative levels of OT and OT-GKR (and, hence, the relative levels of OT-processing enzymes) could have a finely-tuned, regulatory impact on heart development and homeostasis.

### Conclusion

The present study demonstrates that OT-GKR peptides have a cardiomyogenic action via OTRs and also via V1aR. The results raise the possibility that C-terminally extended OT molecules can contribute to heart growth during fetal life, even if the post-translational machinery of OT processing is not completely developed. This cardiac OT-GKR differentiation is important in the development of cell therapies for hearts injured by infarction, to induce the cardiac differentiation of somatic stem cells in diseased adult hearts for their regeneration.

## Materials and Methods

### High pressure liquid chromatography (HPLC) and RIA

Dried acetone-extracted homogenates were dissolved in an aqueous solution of 20% acetonitrile containing 0.1% trifluoroacetic acid and applied to a Vydac 218-TP24 column (5×250 mm) for reverse-phase HPLC (Waters, Milford, MA). The column was eluted with a linear gradient of 20–50% CH3CN/0.1% TFA at a flow rate of 1.2 ml/min. The fractions were collected and lyophilized in a Speed-Vac. Direct RIA [15], after reconstitution of the samples in RIA buffer, ascertained the presence of OT-GKR in HPLC fractions. OT-VA18 antibody was used to measure OT-GKR concentration. Synthetic OT as well as pituitary gland extracts, chromatographed under identical conditions, served as standards [6].

### Molecular docking

MolDock software investigated the interaction of OT molecules with OTR and V1aR [48]. This program makes use of predicted cavities during the docking process and identifies potential ligand-binding modes (see www.molegro.com for details). 3-D models of OT-GKR and OT were constructed with the Biopolymer module of the SYBYL molecular modeling package (Tripos Associates, St. Louis, MO). These 3-D models of activated OTR (OTR Gq11) and human vasopressin V1aR were described previously [38]. The MolDock scoring function served to pre-compute score grids for dock evaluation. Potential binding sites were detected with the grid-based cavity prediction algorithm. The saved conformations for ligand-receptor complexes were subjected to detailed 3-D analysis for interactions at active sites.

### Cell culture and differentiation

P19 cells (Crl-1825) from the American Type Culture Collection (Manassas, VA, www.atcc.org) were cultured as reported elsewhere for parental P19 cells [11]. Green fluorescent protein (GFP)-P19Cl6 cells, a gift from Dr. C. L.Mummery (Hubrecht Laboratory, University Medical Center, Utrecht, Netherlands) were cultured as described for parental P19 cells [12]. For differentiation, 1,000 cells in a volume of 25 µL of complete medium were plated in hanging drops onto the lid of bacteriological grade Petri dishes (10-cm diameter) and incubated at 37°C for 2 days in the absence (non-induced) or presence of inducers: OT (10^–6^ M, Cat. No. H-2510, Peninsula Laboratories, San Carlos, CA, www.penlabs.com), OT-Gly, OT-Gly-Lys, OT-Gly-Lys-Arg (synthesized by the Peptide Synthesis & Protein Sequencing Core Facility of Eastern Quebec, Quebec, Canada), OT antagonist (OTA) [d(CH_2_)_5_^1^,Tyr(Me)^2^,Thr^4^,Orn^8^,Tyr-NH_2_^9^]-vasotocin (10^−6^ M, Cat. No. 4015938.0005, Bachem Americas, Inc, Torrance, CA, www.bachem.com) and selective V1aR antagonist (V1A) d(CH_2_)_5_^1^,Tyr(Me)^2^,Arg^8^)-vasopressin (10^−6^ M, Cat. No. H-5350, Bachem). The base of each culture dish contained phosphate-buffered saline (PBS) to prevent the drops from evaporating. After 2 days of incubation, embryoid bodies (EBs) formed in drops were transferred to bacterial dishes coated with poly (2-hydroxyethyl methacrylate) (Cat. No. P3932, Sigma-Aldrich, Oakville, ON, Canada, www.sigmaaldrich.com) to eliminate cell adhesion to growth surfaces, with fresh differentiation medium added and kept for 3 more days in suspension in the absence (non-induced) or presence of inducers. On the 5^th^ day, EBs were collected and plated in complete α-modified Eagle's minimal essential medium in tissue culture dishes in the absence of inducers (NI) until day 14. The medium was refreshed every 48 h.

To analyze cardiomyogenesis in conditions of endogenous OT-GKR production in stem cells, the OT-GKR-IRES-EGFP construct (prepared as reported previously) [15] was transfected into EC P19 cells with a FuGENE 6 kit (Cat. No. 11814443001, Hoffmann-La Roche Limited, Mississauga, ON, Canada). OT-GKR peptide levels in media secreted from control EBs at day 5 were 211.9±62.5 pg/ml whereas the corresponding values obtained in media from OT-GKR-transfected cells disclosed a significant increment of peptide concentration to 581.3±41.5 pg/ml (n = 5, p<0.05).

### OTR and V1aR silencing by siRNA

OTR siRNA duplexes (sense, 5-GGA CUA CAG CAU AGA AAU A-3; antisense, 5-UAU UUC UAU GCU GUA GUC C-3) targeted to the 21-nucleotide mouse OTR mRNA sequence (CAG GAC TAC AGC ATA GAA ATA). V1aR siRNA duplexes (sense, 5′-CAC UGU UGU UUC UAC ACA ATT-3′; antisense, 5′-UUG UGU AGA AAC AAC AGU GCT-3′) to the 20-nucleotide mouse V1aR mRNA sequence (5′-AGC ACT GTT GTT TCT ACA CAA-3′) and validated siRNA negative controls (scrambled siRNA) were designed commercially (Qiagen, Mississauga, ON, Canada, www.qiagen.com/siRNA Cat No 1027292) and prepared as recommended by the manufacturer. Methodology for siRNA transfection in ES cells was adapted from a previous report [20]. Differentiating P19 cell EBs in suspension were transferred to 24-well plates, followed by transfection with OTR siRNA (Cat. No. SI01367779, Qiagen) and V1aR siRNA (Qiagen Cat No SI02673083) according to the manufacturer's instructions. After 24 h, the cells were re-transfected as before and incubated in differentiation media in the presence or absence of OT-GKR. EB outgrowths were examined for beating activity.

### Immunocytochemistry and microscopic analysis

Cell morphology was examined under a Model IX51 inverted microscope (Olympus, Tokyo, Japan, www.olympus.com) equipped for epifluorescence analysis. Phase contrast micrographs were taken with a Q Imaging QICAM-IR Fast 1394 Digital CCD camera. At day 8, contracting cell colonies in fields of 3 independent samples were counted from the video record with Image J software (National Institutes of Health, Bethesda, MD, www.nih.gov). Immunocytochemistry was performed in P19 cells, as described elsewhere [10]. The cells were incubated in 0.2% gelatin-covered Lab-Tek plates (Cat. No. 177437, Nunc International, Rochester, NY, www.nuncbrand.com). They were fixed in a solution containing 4% paraformaldehyde in 0.1 M PBS, pH 7.4, for 10 min. They were then washed with PBS, permeabilized for 10 min with 0.4% Triton X-100, and labeled with specific antibodies. Anti-sarcomeric alpha-actinin monoclonal antibody, which reacts with skeletal and cardiac muscle actinin (clone EA-53, Cat. No. 096K4774, Sigma Chemical Co.), was used at 1∶800 dilution. Mouse monoclonal antibody IgG anti-dihydropyridine receptor-alpha (DHPRα 20-A, Cat. No. ab2864-100) diluted 1∶200 and anti-OCT-4 rabbit polyclonal antibody (Cat. No. ab1876-100) diluted 1∶200 were both from Abcam, Inc., Cambridge, MA, www.abcam.com). Goat polyclonal myosin light chain-2 ventricular (MLC-2v) (C17. Cat. No. sc-34490) was from Santa Cruz Biotechnology, Inc. Santa Cruz Biotechnology, Santa Cruz, CA. The Texas Red-conjugated rabbit polyclonal anti-mouse IgG (Cat. No. ab6726-1) and sheep anti-rabbit IgG (Cat. No. ab6793) were from Abcam, Inc (www.abcam.com). Goat anti-mouse IgG secondary antibody conjugated to red fluorophore Alexa Fluor 568 (Cat. No. A11004) was from Invitrogen, Life Technologies (Carlsbad, CA). V1aR was detected by goat polyclonal antibody C-20 (from Santa Cruz Biotech. Inc., www.scbt.com sc- 18096 diluted 1∶200. To obtain green fluorescence, the cells were probed with secondary Alexa Fluor 488 labeled donkey anti-goat IgG conjugate (Cat. No. A11055, Invitrogen Life Technologies). For immunohistochemical staining, mouse embryos were fixed with 4% formaldehyde and 0.1% picric acid in 0.1 M PBS (pH 7.4), embedded in paraffin or sectioned using cryostat. 5-µm sections were cut longitudinally and mounted on polylysine-treated slides (Cat. No. P-4981, Esco, Erie Scientific Co., Portsmouth, NH). OTRs were detected by rabbit antibody (Cat. No. RDI-OXYTOCRabrx, RDI Division of Fitzgerald Industries International, Concord, MA, www.researchd.com/miscabs/oxytocr.htm) diluted 1∶700. OT-specific antibodies could be divided into those that only recognized C-terminally amidated forms (OT-MM, a generous gift from Dr. Mariana Morris of Wright State University, Dayton, OH) and those that recognized also C-terminally extended peptides (OT-VA18) characterized in Dr. H. Gainer's Laboratory of Neurochemistry, National Institutes of Health, Bethesda, MD) [1]. Polyclonal rabbit antibody specifically recognizing OT-GKR peptide (VA18, a generous gift from Dr. Gainer), was diluted 1∶700. Staining was revealed by the biotin-streptavidin method with Histostain Plus Rabbit Primary 3,3′-diaminobenzidine (DAB) kit (Cat. No. 85-9243, Zymed Laboratories, San Francisco, CA, www.zymed.com), counterstained with hematoxylin and eosin according to standard procedures. After reaction with anti-OT-GKR antibodies, cryostat cardiac sections were probed with Texas Red-labeled goat anti-rabbit IgG conjugate (1∶200; Cat. No. ab6793, Abcam, Inc.). Monoclonal antibody against cardiac troponin (1∶100, Cat. No. ab7217-7, Abcam, Inc.) was deployed for CM identification. To obtain green fluorescence, secondary biotinylated rabbit antibody against mouse IgG (BA-2001, Horse Vector Laboratories, Burlingame, CA) was followed by streptavidin-Alexa Fluor 488 conjugate (S11223, Invitrogen Life Technologies). Control staining, obtained by overnight pre-incubation of anti-OT-GKR antibody at 4°C in the presence of 10^–6^ M synthetic OT-GKR or the omission of primary antibodies, was negative, emphasizing specificity of immunocytochemistry. Panoramic, cross-sectional, digital images of stained whole embryos were prepared with Adobe Photoshop CS software (Adobe Systems Inc., San Jose, CA).

For transferase dUTP nick end labeling (TUNEL) reaction indicative of apoptosis, the DeadEnd Fluorometric TUNEL System (Cat. No. G3250, Promega, Montreal, QC, Canada) was used.

### Fluorescence-activated cell sorting (FACS) analysis

GFP-P19Cl6 cell culture was digested to single-cell suspension with Accutase (Cat. No. AT104, Innovative Cell Technologies, Inc., San Diego, CA, www.innovativecelltech.com) for adhered cells (days 6 and 14) or Accumax (Cat. No. AM105, Innovative Cell Technologies, Inc.) for suspended EBs (day 5 of differentiation). The dissociated cells were washed with PBS, suspended in PBS containing Ca^2+^ (1 mM) and Mg^2+^ (0.5 mM) at room temperature, and filtered with a cell strainer with 70 µm nylon mesh (Falcon, Cat. No. 352360, BD Biosciences, Mississauga, ON, Canada, www.bdbiosciences.ca). GFP-positive cells were quantified by the passage of minimum 10,000 viable cells and sorted in the FL1 channel on the basis of forward-scattered and side-scattered light in a FACS Aria™ Cell Sorter (BD Biosciences) as the cells traversed the beam of an argon ion laser (488 nm). The BD Biosciences software program CellQuest was applied for data acquisition and analysis. Non-induced cells served as negative controls. Sorted GFP-positive cells were collected in culture media and allowed to reattach to culture dishes, at least for the confirmation of beating contractility.

### Reverse transcription-polymerase chain reaction (RT-PCR)

Total cellular RNA was extracted with TRIzol Reagent (Cat. No. 15596-018, Invitrogen Life Technologies) according to the manufacturer's protocol. To remove genomic DNA, total RNA was treated with 2 units of Turbo DNase (Turbo DNA-free, Cat. No. AM1907, Applied Biosystems/Ambion. Streetsville, ON, Canada). First-strand cDNA was synthesized in a final volume of 40 µl containing first-strand buffer, 4 µg of cellular RNA, 4 µl of hexanucleotide primers (Cat. No. 588753, Invitrogen Life Technologies) and avian myeloblastosis virus reverse transcriptase (12 units/µg RNA, Cat. No. 28025-013, Invitrogen Life Technologies) for 180 min at 37°C. First-strand cDNA (5 µl) was then utilized for PCR amplification with exon-specific oligonucleotide primers in a Robocycler Gradient 40 thermocycler (Stratagene, La Jolla, CA). For all PCR studies, the number of cycles employed was within the linear range of amplification. These values were normalized to corresponding 18S mRNA. Primer sequences and conditions for the PCR analysis of 143-bp GATA-4 NM_008092 (annealing temperature 61°C, 32 cycles), 57-bp Mef2c NM_025282.1 (annealing temperature 60°C, 34 cycles), 272-bp Myogenin NM_008092 (annealing temperature 54°C, 18 cycles) and 144-bp MyoD NM_010866 (annealing temperature 54°C, 30 cycles) mouse transcripts have already been described [12]. The PCR products were size-fractionated by 2% agarose gel electrophoresis and visualized with the Storm 840 Imaging System and ImageQuant software (Version 4.2, Molecular Dynamics Inc., Sunnyvale, CA).

### Statistics

The results are expressed as mean ± SEM. Comparisons between groups were evaluated by 1-way ANOVA, followed by Newman-Keuls multiple comparison test with the PRISM computer program. Statistical significance was taken as p<0.05.
